# A Subdermal Osteochondroma in a Young Girl

**DOI:** 10.1155/2017/8672816

**Published:** 2017-01-04

**Authors:** Heather A. Cole, Hernan Correa, Jonathan G. Schoenecker

**Affiliations:** ^1^Department of Orthopaedics and Rehabilitation, Vanderbilt University Medical Center, 4202 Doctors' Office Tower, 2200 Children's Way, Nashville, TN 37232-9565, USA; ^2^Department of Pathology, Vanderbilt University Medical Center, 4202 Doctors' Office Tower, 2200 Children's Way, Nashville, TN 37232-9565, USA; ^3^Department of Pediatrics, Vanderbilt University Medical Center, 4202 Doctors' Office Tower, 2200 Children's Way, Nashville, TN 37232-9565, USA; ^4^Vanderbilt Center for Bone Biology, Vanderbilt University Medical Center, 4202 Doctors' Office Tower, 2200 Children's Way, Nashville, TN 37232-9565, USA; ^5^Department of Pharmacology, Vanderbilt University Medical Center, 4202 Doctors' Office Tower, 2200 Children's Way, Nashville, TN 37232-9565, USA

## Abstract

Osteochondromas are common benign tumors of cartilage and bone. They are usually found as contiguous bone with a cartilage cap at the end of the growth plate of long bones. Similar to structure are extraskeletal osteochondromas. However, unlike typical osteochondromas, extraskeletal osteochondromas are noncontinuous with bone. To our knowledge, all reported extraskeletal osteochondromas have been contained within fascial compartments. Here we present the case of a 5-year-old female who had a slow growing mass of the anterior distal right thigh. Imaging studies revealed an ossified mass extending from dermal layer of the subcutaneous tissue with no connection to the underlying deep fascia. An excisional biopsy was performed and proved to be a subdermal extraskeletal osteochondroma.

## 1. Introduction

An osteochondroma by definition is a benign tumor that includes components of both cartilage and bone [1–4]. A conventional osteochondroma occurs in the metaphysis of a long bone, is continuous with the adjacent bone, and extends into the soft tissues led by a proliferative cartilaginous cap. In reality, an osteochondroma can occur anywhere in which a population of undifferentiated chondrocytes exists, and there have been numerous reports of extraskeletal osteochondromas [1–17]. These rare, benign soft-tissue lesions must be differentiated from other mineralizing lesions, such as myositis ossificans, and more aggressive lesions such as synovial chondromas and synovial sarcomas. To our knowledge, all reported extraskeletal osteochondromas have been contained within fascial compartments. Here we present a case of a subcutaneous osteochondroma in a young girl.

## 2. Case Presentation

A 5-year-old female was seen at clinic, with a chief complaint of a mass in the anterior distal right thigh. The mass was noted to be slow growing but unknown when it first appeared. There were no complaints of pain, no history of trauma in the region, and no other lesions identified. On physical examination, the mass was firm and well adhered to the dermis but freely mobile over the underlying fascia. There were no skin changes or discoloration and the knee exam was otherwise normal.

**Figure 1:**
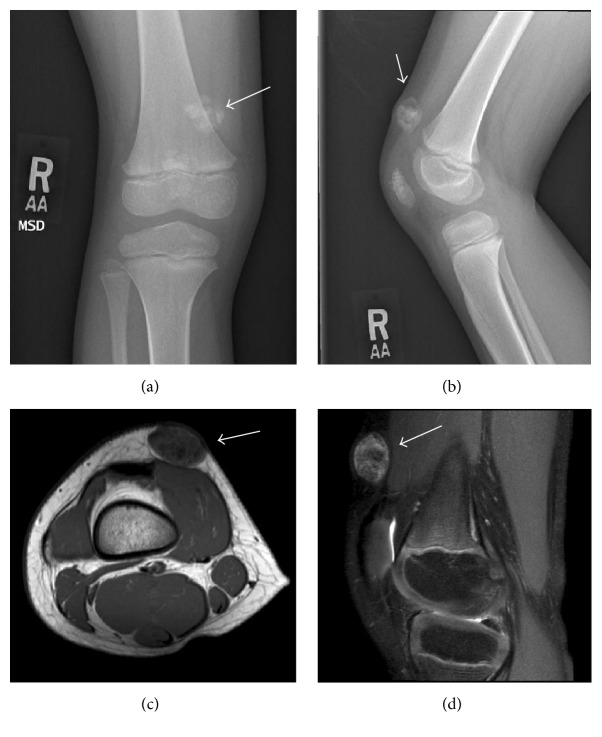
AP (a) and lateral (b) radiographic imaging of distal femur demonstrates aberrant calcification of the suprapatellar region within the soft tissue. Transverse (c) and lateral magnetic resonance imaging (d) reveals that the calcified mass is confined to the subcutaneous tissue with no connections to the underlying bone.

Plain radiographs showed an oval, calcified mass within the soft tissue of the suprapatellar region (Figures 1(a) and 1(b)). Magnetic resonance imagining revealed a mass confined to the subcutaneous soft tissues along the anteromedial aspect of the thigh superficial to the vastus medialis causing extrinsic mass-effect without deep invasion (Figures 1(c) and 1(d)). This mass measured 2.0 cm medial-lateral by 1.4 cm anteroposterior by 1.9 cm cranial caudal with the underlying musculature appearing normal. This mass was predominantly low signal intensity both eccentrically and centrally on all imaging sequences. These findings were considered to show an extraskeletal osteochondroma and an excisional biopsy was discussed with the family.

**Figure 2:**
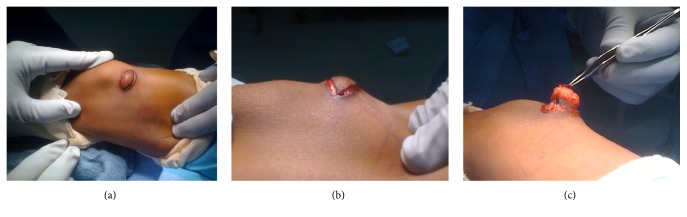
Intraoperative imagining demonstrates the mass within the subcutaneous tissue in the anteromedial region of the distal femur (a). An elliptical incision was made (b) and the calcified mass was carefully dissected and removed (c).

**Figure 3:**
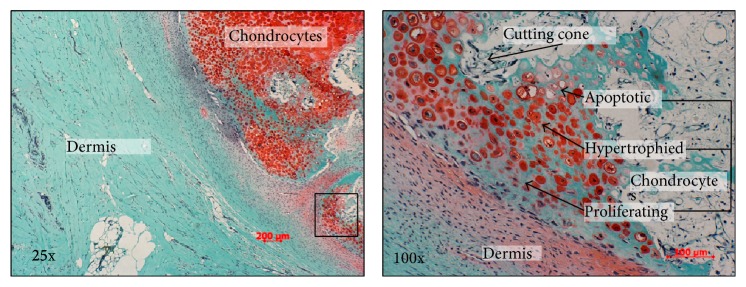
Safranin-O (25x) demonstrates cartilage cap with endochondral bone formation underlying the dermis. Zone of proliferation, hypertrophy, and apoptosis with new bone formation are identified at 100x. These findings are consistent with osteochondroma.

**Figure 4:**
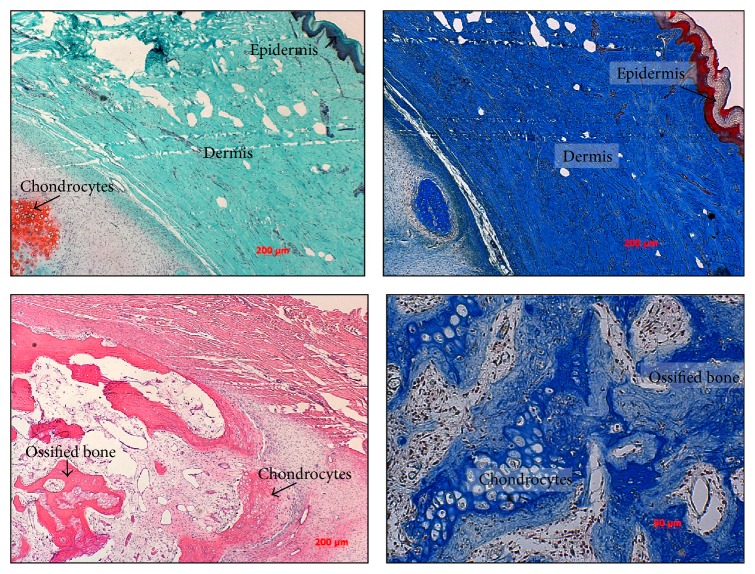
Safranin-O staining (25x) demonstrates calcified chondrocytes located just deep to the dermal area of the tissue (red). Masson's Trichrome (25x) demonstrates the epidermal (red) and dermal (blue) layer that is directly adjacent to the chondrocytic area. H&E and Masson's Trichrome (25x) demonstrate endochondral bone formation adjacent to a cartilaginous cap indicating the formation of an osteochondroma.

For the excision biopsy, an elliptical incision was made surrounding the nonadhesive area of the skin in Langer's lines (Figure 2). The mass was then dissected circumferentially and easily removed as there were no attachments to the deep fascia. Grossly, the specimen was hard, tan-pale pink, bony cut surface with multifocal areas of firm, white, glistening, cartilaginous tissue. No discrete areas of hemorrhage or necrosis were grossly identified. A representative portion of tissue was submitted for histopathology. The sections demonstrated a nodular, slightly disorganized cartilaginous cap surrounding the lesion that progressed to endochondral-like bone formation (Figures 3 and 4). No evidence of malignancy was identified. No reoccurrence had developed at last follow-up clinic visit at age 9. No photographs or radiographs were available.

## 3. Discussion

Osteochondromas are the most common benign tumors occurring in the metaphysis of long bones [1, 2, 5]. Most commonly they present as an extension of the metaphysis consisting of trabecular bone with a cartilaginous cap. Germline mutation and functional loss of EXT1 or EXT2 are commonly found in multiple osteochondromas [6]. These proteins are expressed in the growth plate and it is known that mutations of these proteins can lead to heparin sulfate deficiency [6]. Heparin sulfate is an essential component of cell surface and matrix-associated proteoglycans and significant in regulating distribution and activity of signaling proteins [6]. Therefore, aberrant signaling factors are thought to lead to exostosis formation [6].

Rarely are these lesions extraskeletal or independent of the bone, but they have been reported in the hand, feet [3, 7, 8, 10–12], knee [13–15], and hip [16, 17], most commonly near tendon sheaths, joint capsule, or periosteum and most commonly found within the deep fascia. It has been suggested that the pathogenesis of such tumors occurs through either cellular migration, precartilaginous tissue persisting in the tendon, or metaplasia of mesenchymal cells or fibroblasts. Here we show for the first time to our knowledge as searched from English literature that osteochondroma extended from the dermal layer of the skin with complete containment within the subcutaneous tissue.

When presented with a well-circumscribed calcified lesion various possible differential diagnoses should be considered. These include myositis ossificans, lipomatous lesion, tumoral calcinosis, synovial sarcoma, and extraskeletal osteosarcoma, the most common being myositis ossificans. This diagnosis was considered initially with radiographic and magnetic resonance imaging. However, this lesion was confirmed by histopathology with the presence of trabecular bone surrounded by a cartilaginous cap and fibrous capsule (Figure 3). This is distinctly different than myositis ossificans, which is characterized by a zonal phenomenon of peripheral calcification and able to change size within a few weeks. While we acknowledge that extraskeletal osteochondromas are rare, the purpose of this paper is to present awareness to alternative diagnoses to circumscribed, calcified lesions.
